# The Causal Relationship between PCSK9 Inhibitors and Malignant Tumors: A Mendelian Randomization Study Based on Drug Targeting

**DOI:** 10.3390/genes15010132

**Published:** 2024-01-21

**Authors:** Wenxin Wang, Wei Li, Dan Zhang, Yongrun Mi, Jingyu Zhang, Guoyang He

**Affiliations:** 1 Department of Pathology, Xinxiang Medical University, Xinxiang 453003, China; wangwenxinisme@163.com (W.W.); shuiyao112112@163.com (D.Z.); zjingyu233@outlook.com (J.Z.); 2 School of Forensic Medicine, Xinxiang Medical University, Xinxiang 453003, China; lwhn2008@163.com (W.L.); miyongrun@outlook.com (Y.M.)

**Keywords:** PCSK9 inhibitor, drug target, Mendelian randomization, tumor

## Abstract

**Objective:** This study explores the potential causal association between proprotein convertase subtilisin/kexin 9 (PCSK9) inhibitors and tumor development using Mendelian randomization (MR) based on drug targets. **Methods:** Instrumental variables within ±100 kb of the *PCSK9* gene locus, impacting low-density lipoprotein cholesterol (LDL-C), were utilized for MR analysis. Coronary heart disease (CHD) served as a positive control to validate the causal relationship between PCSK9 inhibitors and various cancers. We employed reverse MR to address the reverse causation concerns. Data from positive controls and tumors were sourced from OpenGWAS. **Results:** MR analysis suggested a negative causal relationship between PCSK9 inhibitors and both breast and lung cancers (95%*CI*_Breast cancer_ 0.81~0.99, *p* = 2.25 × 10^−2^; 95%*CI*_Lung cancer_ 0.65~0.94, *p* = 2.55 × 10^−3^). In contrast, a positive causal link was observed with gastric, hepatic, and oral pharyngeal cancers and cervical intraepithelial neoplasia (95%*CI*_Gastric cancer_ 1.14~1.75, *p* = 1.88 × 10^−2^; 95%*CI*_Hepatic cancer_ 1.46~2.53, *p* = 1.16 × 10^−2^; 95%*CI*_Oral cavity and pharyngeal cancer_ 4.49~6.33, *p* = 3.36 × 10^−4^; 95%*CI*_Carcinoma in situ of cervix uteri_ 4.56~7.12, *p* = 6.91 × 10^−3^), without heterogeneity or pleiotropy (*p* > 0.05). Sensitivity analyses confirmed these findings. The results of MR of drug targets suggested no causal relationship between PCSK9 inhibitors and bladder cancer, thyroid cancer, pancreatic cancer, colorectal cancer, malignant neoplasms of the kidney (except for renal pelvis tumors), malignant neoplasms of the brain, and malignant neoplasms of the esophagus (*p* > 0.05). Reverse MR helped mitigate reverse causation effects. **Conclusions:** The study indicates a divergent causal relationship of PCSK9 inhibitors with certain cancers. While negatively associated with breast and lung cancers, a positive causal association was observed with gastric, hepatic, oral cavity, and pharyngeal cancers and cervical carcinoma in situ. No causal links were found with bladder, thyroid, pancreatic, colorectal, certain kidney, brain, and esophageal cancers.

## 1. Introduction

The World Health Organization reports that tumors remain the leading cause of global mortality [1]. Despite advancements in cancer research, significant treatment challenges persist [2]. Cholesterol, essential for life, is balanced by synthesis, uptake, efflux, and esterification [3]. Research, including that of Revilla et al., highlights the key role of low-density lipoprotein (LDL) in various tumors [4]. Elevated extracellular levels of LDL-C have been linked to the proliferation of prostate cancer cells [5,6], though studies on their correlation with overall prostate cancer risk are inconsistent [7,8,9,10]. Prospective studies have shown that LDL-C plays a significant role in breast cancer progression. LDL-C is also significantly implicated in breast cancer progression, however Martin et al. reported a negative association between LDL-C levels and breast cancer risk [11].

Statins, effective in lowering LDL-C levels, have shown significant anticancer properties. These include anti-proliferative, pro-apoptotic, and anti-invasive characteristics across various cancers. In vitro and in vivo studies on colon cancer, pancreatic cancer, and melanoma demonstrate the inhibitory effects of statins on tumor progression. For instance, lovastatin influences lung cancer cells through several pathways, including COX-2 upregulation and PPARγ activation, leading to apoptosis. It also inhibits growth and induces cytotoxicity in gefitinib-resistant non-small cell lung cancer cells, likely through cleavage of caspase-3, PARP, and Bax. Atorvastatin shows anti-tumor effects and potentially reduces the risk of lung cancer. Simvastatin regulates A549 cells, impeding cancer development through oxidative stress metabolites and SOD2 expression. Fluvastatin induces cell cycle arrest and apoptosis, upregulating p21 and p53, thereby achieving anti-tumor effects. Various statins inhibit tumor growth through mechanisms such as cell cycle arrest, apoptosis induction, antioxidant defense, and PI3K/AKT/mTOR pathway activation, demonstrating their promising anticancer potential despite some controversial findings [12,13].

PCSK9, a serine protease regulating LDL-C metabolism, has emerged as a significant target for cholesterol-lowering therapy [14]. Its role, notably in colorectal cancer, breast cancer, and lymphoblastic leukemia, is of growing interest due to its upregulation and association with poor prognosis [15,16,17,18,19]. PCSK9 inhibitors may enhance the efficacy of cancer treatment [20,21,22,23,24], although their function in liver cancer remains unresolved [25]. Emerging evidence from these studies suggests that PCSK9 inhibitors might not have a universal effect against various tumors, indicating a limited spectrum in their antitumor efficacy.

This study employed MR of drug targets to simulate pharmacological inhibitory effects using genetic variation as an instrumental variable. Through the analysis of genome-wide association studies (GWAS), this study probed the causal link between PCSK9 inhibitors and cancer. Our aim was to enhance the understanding of the role of PCSK9 inhibitors in oncogenesis and to provide theoretical insights for their clinical application in cancer therapeutics.

## 2. Materials and Methods

### 2.1. Study Design

**Figure 1:**
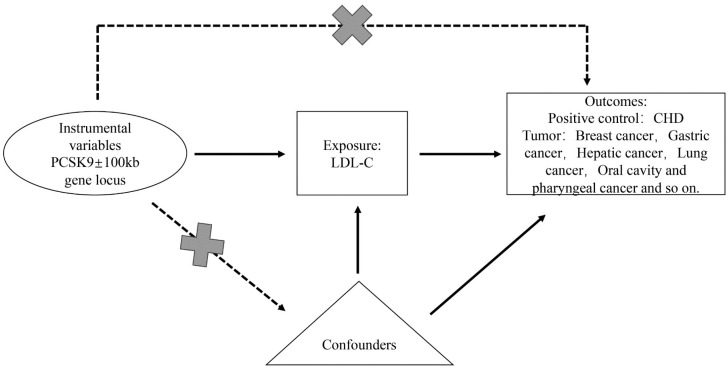
Overview and design of Mendelian randomization analysis for drug target PCSK9 inhibition.

Based on Mendelian randomization two-sample analysis, we investigated the causal relationship between single nucleotide polymorphisms (SNPs) within ±100 kb of the *PCSK9* gene locus, which is closely associated with low-density lipoprotein cholesterol (LDL-C) and various malignant tumors (breast cancer, lung cancer, gastric cancer, hepatic cancer, oral cavity and pharyngeal cancer, carcinoma in situ of the cervix uteri, bladder cancer, thyroid cancer, pancreatic cancer, colorectal cancer, malignant tumors of the kidney excluding the renal pelvis, malignant brain tumors, and esophageal malignant tumors). Mendelian randomization utilizes genetic variants as proxies for risk factors; hence, effective instrumental variables (IVs) in causal inference must satisfy three key assumptions: (1) genetic variants are directly associated with exposure; (2) genetic variants are unrelated to confounding factors that might exist between exposure and outcome; (3) genetic variants do not affect the outcome through pathways other than exposure (Figure 1).

### 2.2. Selection of Instrumental Variables

The selection of PCSK9 instrumental variables was based on the latest LDL-C summary data from OpenGWAS (https://gwas.mrcieu.ac.uk/, accessed on 16 September 2023), which included 201,678 European individuals. We selected these instrumental variables to target the reduction in LDL-C by PCSK9 and to simulate the effects of PCSK9 inhibitors [26]. The instrumental variable selection was situated within a ±100 kb region around the PCSK9 gene locus and was closely associated with LDL-C (*p* < 5 × 10^−8^, Figure 1). To remove the effect of linkage disequilibrium (LD) on the results, the LD threshold was set to r^2^ < 0.3, and 16 significant SNPs in PCSK9 were retained. Using another set of LDL-C summary data from OpenGWAS, which included 440,546 Europeans, the same method was applied to obtain instrumental variables for PCSK9 analysis, ensuring result stability and consistency.

### 2.3. Sources of Outcome Data

The data for the 14 types of tumors mentioned above were obtained from OpenGWAS and are based on the European population. The positive control dataset was coronary heart disease (CHD), which contained 22,233 cases and 64,762 controls. Tumor data included breast cancer, gastric cancer, hepatic cancer, lung cancer, oral cavity and pharyngeal cancer, carcinoma in situ of the cervix uteri (endocervix), bladder cancer, thyroid cancer, pancreatic cancer, colorectal cancer, malignant neoplasm of the kidney (except for renal pelvis tumors), malignant neoplasm of the brain, and malignant neoplasm of the esophagus. Neither exposure nor outcome used data from the same institution, and there was no data overlap.

### 2.4. Reverse Mendelian Randomization Validation

Positive controls (CHD) and tumor data (including breast cancer, gastric cancer, hepatic cancer, lung cancer, oral cavity and pharyngeal cancer, carcinoma in situ of the cervix uteri (endocervix), bladder cancer, thyroid cancer, pancreatic cancer, colorectal cancer, malignant neoplasm of the kidney (except renal pelvis), malignant neoplasm of the brain, and malignant neoplasm of the esophagus) were employed as exposures, with LDL-C serving as the outcome in a reverse two-sample Mendelian randomization analysis. The SNP selection threshold for each exposure was set at (5 × 10^−6^, clump = 10,000 kb, r^2^ < 0.001). Afterwards, we used another set of LDL-C data to validate the reverse MR results, and the SNP screening conditions were the same as before.

### 2.5. Data Analysis

MR-Egger, weighted median, IVW, Simple mode, and weighted mode methods were used to analyze exposure-related drug-targeted instrumental variables and outcome data sets, and the IVW method is most commonly used [27]. Heterogeneity testing was performed using the MR-Egger and IVW methods, and the Cochrane Q value was used to evaluate the heterogeneity of the genetic tools. Q > 0.05, indicating no heterogeneity. The horizontal pleiotropy of the genetic tools was assessed using the MR-Egger regression equation, with *p* > 0.05, indicating the absence of horizontal pleiotropy [28].

The MR analysis assumes that the SNP associated with exposure is not directly related to the outcome and is free from confounding factors (Figure 1). The online platform PhenoScanner (http://www.phenoscanner.medschl.cam.ac.uk/, accessed on 18 September 2023) was used to identify traits directly associated with instrumental variable SNPs and to exclude SNPs associated with the outcome and confounders. To ensure that the results were not significantly influenced by individual SNPs, we performed a leave-one-out analysis by sequentially removing each SNP and comparing the results of the IVW method with those of all variants. Data analysis was conducted using the MR-PRESSO and TwoSampleMR packages in R version 4.2.1.

## 3. Result

### 3.1. Causal Relationship between PCSK9 Inhibitors and CHD

**Figure 2:**
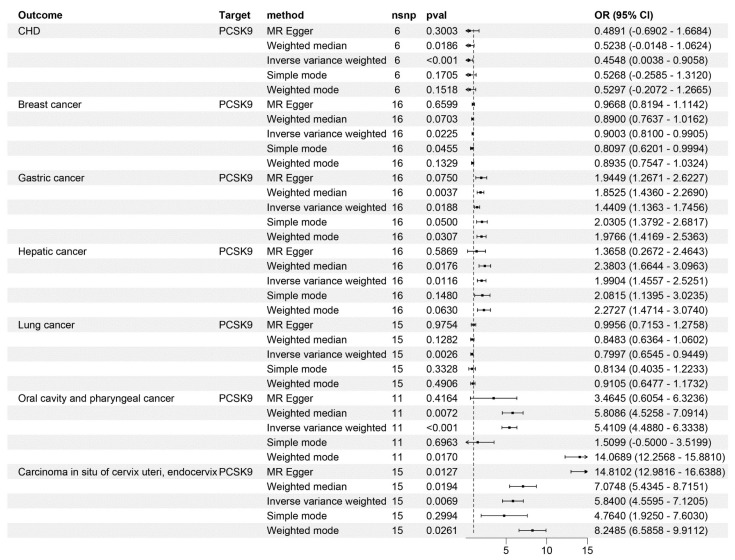
Mendelian randomization analysis results for drug targets. The causal relationship between PCSK9 inhibitors and CHD, breast cancer, gastric cancer, hepatic cancer, lung cancer, oral cavity and pharyngeal cancer, and carcinoma in situ of the cervix uteri and endocervix. nsnp, number of nucleotide polymorphisms; OR, odds ratio; CI, confidence interval.

Due to the fact that PCSK9 inhibitors have become therapeutic drugs for CHD, we used CHD as a positive control to validate the effectiveness of the instrumental variable. The IVW method consistently demonstrated that PCSK9 inhibitors significantly lower CHD risk. (OR = 0.45, 95%*CI*: 0.003–0.91, *p* = 6.17 × 10^−4^, Figure 2). This finding was corroborated by repeated analyses using an alternative LDL-C dataset (OR = 0.412, 95%*CI*: 0.035–0.859, *p* = 1.05 × 10^−4^, Table 1), reinforcing the conclusion that PCSK9 inhibitors demonstrate a substantial effect in reducing CHD risk.

### 3.2. Causal Relationship between PCSK9 Inhibitors and Tumors

**Figure 3:**
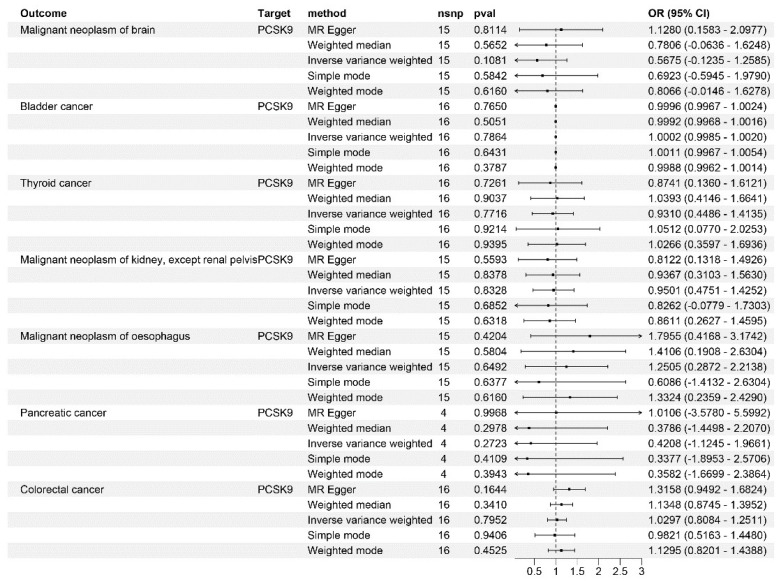
Mendelian randomization analysis results for drug targets. The causal relationship between PCSK9 inhibitors and bladder cancer, thyroid cancer, pancreatic cancer, colorectal cancer, and malignant tumors of the brain, kidney (except for renal pelvis tumors), and esophagus.

In our IVW analyses, PCSK9 inhibitors showed significant protective effects against breast and lung cancers (OR = 0.9, 95%*CI*_breast cancer_: 0.81~0.99; *p* = 2.25 × 10^−2^; OR = 0.79, 95%*CI*_lung cancer_: 0.65~0.94, *p* = 2.55 × 10^−3^, Figure 2). However, the same IVW analyses suggested an increased risk of developing gastric, hepatic, and oral cavity and pharyngeal cancers, and carcinoma in situ of the cervix uteri and endocervix associated with PCSK9 inhibitor use (OR = 1.44, 95%*CI* _gastric cancer_: 1.14~1.75, *p* = 1.88 × 10^−2^; OR = 1.99, 95%*CI*_hepatic cancer_: 1.46~2.53, *p* = 1.16 × 10^−2^; OR = 5.41, 95%*CI*_oral cavity and pharyngeal cancer_: 4.49~6.33, *p* = 3.36 × 10^−4^; OR = 5.84, 95%*CI*_Carcinoma in situ of cervix uteri_: 4.56~7.12, *p* = 6.91 × 10^−3^, Figure 2). Notably, no causal relationship was observed between PCSK9 inhibitors and the risk of bladder (*p* = 0.786), thyroid (*p* = 0.771), pancreatic (*p* = 0.282), and colorectal cancers (*p* = 0.795), and kidney (excluding renal pelvis tumors) (*p* = 0.832), brain (*p* = 0.108), and esophageal malignancies (*p* = 0.649) (Figure 3). These findings were consistent across different LDL-C datasets (Table 1).

### 3.3. Sensitivity, Heterogeneity, and Horizontal Pleiotropy Analysis

**Figure 4:**
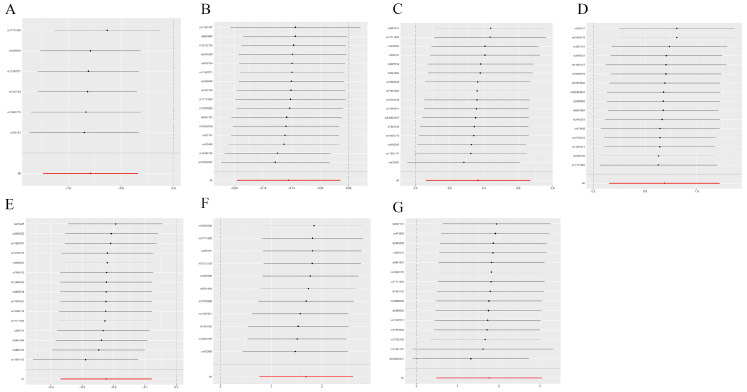
Sensitivity analysis. The comprehensive effect of the remaining SNPs was consistent with the main effect of removing one SNP. (**A**) CHD; (**B**) breast cancer; (**C**) gastric cancer; (**D**) hepatic cancer; (**E**) lung cancer; (**F**) oral cavity and pharyngeal cancer; (**G**) carcinoma in situ of the cervix uteri and endocervix.

**Figure 5:**
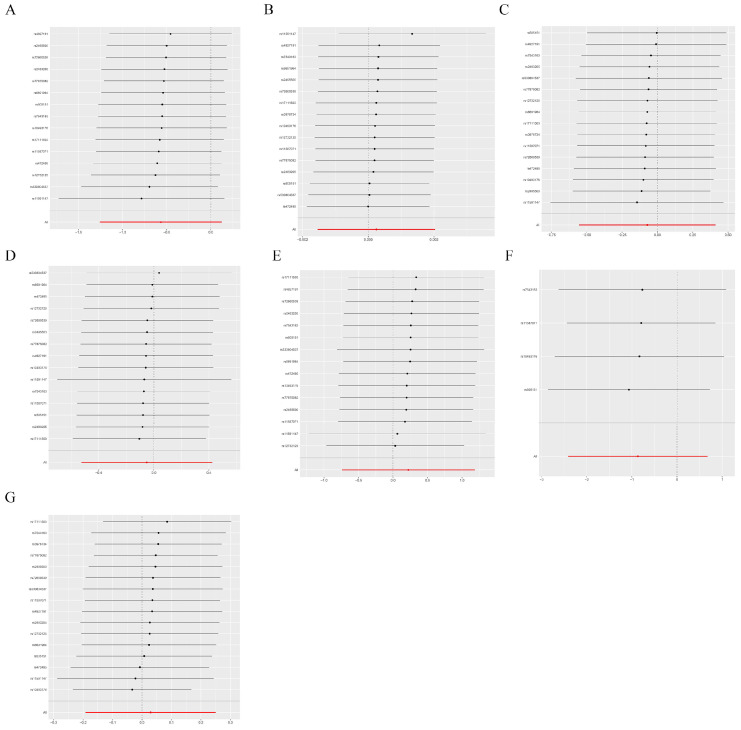
Sensitivity analysis. If the comprehensive effect of the remaining SNPs is consistent with the main effect after removing one SNP. (**A**) Malignant neoplasm of brain, (**B**) Bladder cancer, (**C**) Thyroid cancer, (**D**) Malignant neoplasm of kidney, except renal pelvis, (**E**) Malignant neoplasm of esophagus, (**F**) Pancreatic cancer, (**G**) Colorectal cancer.

We employed Cochrane’s Q and MR-Egger regression analyses to evaluate heterogeneity and horizontal pleiotropy in our outcomes. These analyses revealed no outliers and provided no evidence of heterogeneity or horizontal pleiotropy across all outcomes (Table 2, *p* > 0.05). Furthermore, a leave-one-out analysis affirmed the robustness of our results, showing that the removal of any single SNP did not significantly alter the findings (Figure 4 and Figure 5). This consistency was also observed when replicating the analysis with alternative datasets (Table 3).

### 3.4. Reverse Mendelian Randomization Validation

Reverse MR analysis was utilized to address potential reverse causality, ensuring the robustness of our results. This analysis was conducted to investigate the causal relationships between the positive control (CHD) and LDL-C levels, as well as between various malignant tumors and LDL-C levels. The findings indicated no causal relationship between CHD and LDL-C levels (Table 4, *p* > 0.05). Similarly, there was no causal relationship between various malignant tumors and LDL-C levels, as well as no causal link between a range of malignant tumors and LDL-C levels (Table 4, *p* > 0.05). This absence of causality was further confirmed by validating the results with a secondary dataset (Table 4 and Table 5, *p* > 0.05).

## 4. Discussion

PCSK9 is integral to lipid homeostasis, primarily through modulating LDL cholesterol, which is crucial in neuronal apoptosis [29]. The emergence of PCSK9 inhibitors, aimed at reducing LDL levels, has marked a significant advancement in CHD prevention and treatment [30,31]. Recent studies have highlighted the substantial role that cholesterol plays in tumor development and progression. For instance, tumor-induced hyperlipidemia can disrupt hepatic lipoprotein homeostasis, leading to increased LDL-C levels. Given their influence over LDL [32], PCSK9 inhibitors may thus play a crucial role in the development of tumors.

Our analysis revealed that PCSK9 inhibitors significantly lower the risk of breast and lung cancers. In a plasma study of breast cancer patients, Emilie et al. reported elevated PCSK9 levels in breast cancer patients compared to those with benign lesions [21]. Similarly, Pseurotin A showed a strong inhibitory effect on breast cancer progression and local recurrence induced by high cholesterol after lowering PCSK9 secretion and blocking PCSK9-LDLR binding [33]. However, the reported influence of LDL-C on breast cancer varies across studies, promoting the proliferation and migration of ER- cell lines but not in ER+ cells [4]. Luo et al. linked high PCSK9 expression with poor prognosis in lung adenocarcinoma, noting that PCSK9 inhibitors might counter this by inducing mitochondrial dysfunction in lung cancer cells [34]. This aligns with our findings. Marimuthu et al. reported the high expression of PCSK9 in gastric cancer [35], linked to increased invasion, metastasis, and poor patient prognosis [19]. Studies conducted by Alannan et al. highlighted the ferroptosis-mediated metabolic exhaustion anticancer mechanisms of PCSK9 inhibitors using an in vivo model [36]. He et al. found that downregulation of PCSK9 could exacerbate the progression of liver cancer [37]. There are currently no reports on the role of PCSK9 in oral cavity and pharyngeal cancers or carcinoma in situ of the cervix uteri or endocervix. Yet, our findings suggest PCSK9 inhibitors as potential risk factors for these cancers. Furthermore, we found no evidence linking PCSK9 inhibitors to bladder, thyroid, pancreatic, colorectal cancers, kidney malignancies (excluding renal pelvis tumors), brain, or esophageal cancers, reinforcing the non-association of PCSK9 with these seven types of cancer.

Our findings related to breast, gastric, and lung cancers and hepatocellular carcinoma show some variation from prior studies. We believe that these differences are primarily due to factors such as ethnicity, age, tumor subtypes, and sample sizes. Specifically, in breast cancer, the lack of subtype classification (e.g., Luminal A, Luminal B, Triple-negative/basal-like, and HER2-enriched) could explain the discrepancies. In gastric cancer research, limited studies have been published and variations in ethnicity and sample size might account for the differences. Ioannou and colleagues suggested that disparities in liver cancer outcomes may stem from varying cholesterol levels in the liver [25]. In another MR study [38], inconsistent findings regarding the impact of PCSK9 inhibitors on tumors, particularly gastric cancer, lung cancer, and hepatocellular carcinoma, were noted. We attribute these differences to two factors: (1) our study used more recent and larger LDL data sets from 2020 and 2022, unlike previous studies that relied on 2013 data, and (2) we employed an additional LDL data set for validation and rigorously excluded reverse causality effects, enhancing the robustness of our results.

Currently, two distinct types of PCSK9 inhibitors are recognized, each with a unique mechanism of action [39]: (1) a small interfering RNA (siRNA), which silences the PCSK9 gene, thereby inhibiting its synthesis, and (2) monoclonal antibodies or mimetic antibody proteins, which prevent PCSK9 protein from binding to LDL-R. Both inhibitor types aim to diminish the function of PCSK9, potentially suppressing tumor growth. However, there is no research yet comparing the efficacy of these two inhibitor classes in tumor treatment [39].

In this study, we explored the relationship between PCSK9 inhibitors and tumors. PCSK9 is crucial in regulating LDL-C metabolism, and long-term use of its inhibitor in tumor treatment may lead to a decrease in LDL-C below the normal range [40]. Regular monitoring of LDL-C levels is thus essential when employing long-term PCSK9 inhibition.

To ensure the scientific rigor and consistency of our MR analysis, our approach adopted several key measures. Firstly, we examined the genetic-level relationship between PCSK9 inhibitors and tumor development, effectively mitigating the impact of confounding variables. Secondly, precise SNP, rigorous outliers detection, and the use of CHD as a positive control were instrumental in ensuring the reliability of our findings. Thirdly, the application of five distinct MR methodologies further strengthened the robustness of our results, supplemented by comprehensive evaluations of heterogeneity, horizontal pleiotropy, and sensitivity. Importantly, we also accounted for potential reverse causation effects. Our findings not only enhance our understanding of the role of PCSK9 inhibitors in tumor therapy but also provide a theoretical basis for their clinical application.

In future research, functional screening methods and the exploration of genetic determinants influencing drug responses will be crucial. High-throughput genetic experiments can yield insights into phenotype proliferation or transcriptome changes. Utilizing data from TCGA, DepMap Portal, and cBioPortal will be particularly beneficial. Advanced methods such as 3D organ-like models and patient-derived xenotransplantation offer biologically relevant platforms. These approaches can provide new evidence for the application of PCSK9 inhibitors in treating malignant tumors [41].

The aim of big data analysis is patient-centric; while acknowledging the strengths of MR we recognize its inability to supplant clinical trials. Looking ahead, it is imperative to focus on the practical applications of large data by integrating them with rigorous clinical trials. This approach has the potential to substantially benefit both patient health and the broader field of medical practice. Further, categorizing big data based on specific parameters such as ethnicity, age, and tumor subtypes offers a promising direction for research. Finally, refining the MR algorithm is crucial to augment the accuracy and reliability of large-scale data analyses.

## 5. Conclusions

In summary, we demonstrated the causal relationship between gene-mediated PCSK9 inhibitors and malignant tumors through comprehensive dual-sample MR analysis, emphasizing the model of causal relationship between the immune system and malignant tumors.

## Figures and Tables

**Table 1:** IVW results from repeated analysis using an alternative set of data.

Outcome	Method	nsnp	pval	or	or_lci95	or_uci95
CHD	IVW	9	0.000105	0.412278	0.03543	0.859984
Breast cancer	IVW	33	0.046465	0.915973	0.829577	1.002369
Gastric cancer	IVW	33	0.009436	1.466226	1.177273	1.755179
Hepatic cancer	IVW	33	0.031913	1.748559	1.238079	2.259039
Lung cancer	IVW	29	0.001287	0.770876	0.612424	0.929328
Oral cavity and pharyngeal cancer	IVW	26	4.74 × 10^−5^	12.95632	11.7222	14.19044
Carcinoma in situ of cervix uteri, endocervix	IVW	29	0.006413	6.024705	4.733405	7.316005
Malignant neoplasm of brain	IVW	29	0.13796	0.584619	0.12463	1.293866
Bladder cancer	IVW	33	0.817092	1.000201	0.9985	1.001901
Thyroid cancer	IVW	33	0.640864	1.11934	0.645657	1.593023
Malignant neoplasm of kidney, except renal pelvis	IVW	29	0.806225	0.941211	0.457094	1.425327
Malignant neoplasm of esophagus	IVW	29	0.613284	1.284654	0.31321	2.256097
Pancreatic cancer	IVW	4	0.246173	0.343108	−1.46481	2.151024
Colorectal cancer	IVW	33	0.703725	1.039738	0.838898	1.240578

**Table 2:** Outliers, heterogeneity, and pleiotropy analyses.

Outcome	Heterogeneity Test (Q-Value)		Pleiotropy Test (*p*-Value)	Outlier Test (*p*-Value)
	MR-Egger	IVW		
CHD	0.89	0.81	0.25	NA
Breast cancer	0.49	0.46	0.25	NA
Gastric cancer	0.74	0.74	0.35	NA
Hepatic cancer	0.73	0.75	0.45	NA
Lung cancer	0.72	0.60	0.08	NA
Oral cavity and pharyngeal cancer	0.75	0.61	0.70	NA
Carcinoma in situ of cervix uteri, endocervix	0.87	0.79	0.18	NA
Malignant neoplasm of brain	0.63	0.41	0.073	NA
Bladder cancer	0.97	0.98	0.55	NA
Thyroid cancer	0.97	0.98	0.82	NA
Malignant neoplasm of kidney, except renal pelvis	0.85	0.87	0.56	NA
Malignant neoplasm of esophagus	0.82	0.84	0.48	NA
Pancreatic cancer	0.97	0.97	0.72	NA
Colorectal cancer	0.25	0.17	0.13	NA

**Table 3:** Analysis of outliers, heterogeneity, and pleiotropy from repeated validation using an alternative set of data.

Outcome	Heterogeneity Test (Q-Value)		Pleiotropy Test (*p*-Value)	Outlier Test (*p*-Value)
	MR-Egger	IVW		
CHD	0.76	0.87	0.90	NA
Breast cancer	0.71	0.75	0.85	NA
Gastric cancer	0.67	0.70	0.61	NA
Hepatic cancer	0.60	0.65	0.92	NA
Lung cancer	0.12	0.13	0.44	NA
Oral cavity and pharyngeal cancer	0.60	0.63	0.48	NA
Carcinoma in situ of cervix uteri, endocervix	0.90	0.86	0.14	NA
Malignant neoplasm of brain	0.49	0.36	0.075	NA
Bladder cancer	0.99	0.99	0.62	NA
Thyroid cancer	0.98	0.99	0.79	NA
Malignant neoplasm of kidney, except renal pelvis	0.42	0.45	0.52	NA
Malignant neoplasm of esophagus	0.69	0.60	0.11	NA
Pancreatic cancer	0.96	0.95	0.99	NA
Colorectal cancer	0.19	0.21	0.51	NA

**Table 4:** Reverse Mendelian randomization results.

Exposure	Method	nsnp	pval	or	or_lci95	or_uci95
CHD	IVW	41	0.373	1.029	0.966	1.095
Breast cancer	IVW	240	0.978	0.998	0.987	1.013
Gastric cancer	IVW	24	0.453	0.996	0.985	1.006
Hepatic cancer	IVW	23	0.149	0.989	0.974	1.003
Lung cancer	IVW	53	0.554	0.994	0.976	1.012
Oral cavity and pharyngeal cancer	IVW	16	0.229	1.005	0.996	1.014
Carcinoma in situ of cervix uteri, endocervix	IVW	6	0.773	1.000	0.996	1.004
Malignant neoplasm of brain	IVW	10	0.983	0.999	0.995	1.003
Bladder cancer	IVW	25	0.482	2.195	0.244	19.730
Thyroid cancer	IVW	13	0.683	0.998	0.989	1.007
Malignant neoplasm of kidney, except renal pelvis	IVW	15	0.488	1.002	0.994	1.010
Malignant neoplasm of esophagus	IVW	8	0.221	0.997	0.993	1.001
Pancreatic cancer	IVW	3	0.352	1.040	0.956	1.132
Colorectal cancer	IVW	69	0.364	1.009	0.989	1.028

**Table 5:** Results of reverse Mendelian randomization using an alternative set of data.

Exposure	Method	nsnp	pval	or	or_lci95	or_uci95
CHD	IVW	41	0.113	1.053	0.987	1.124
Breast cancer	IVW	240	0.728	1.002	0.990	1.013
Gastric cancer	IVW	24	0.944	0.999	0.990	1.008
Hepatic cancer	IVW	23	0.067	0.989	0.978	1.000
Lung cancer	IVW	53	0.949	1.000	0.984	1.016
Oral cavity and pharyngeal cancer	IVW	16	0.133	1.006	0.997	1.015
Carcinoma in situ of cervix uteri, endocervix	IVW	6	0.603	1.000	0.997	1.004
Malignant neoplasm of brain	IVW	10	0.606	0.999	0.996	1.002
Bladder cancer	IVW	25	0.339	2.022	0.477	8.575
Thyroid cancer	IVW	13	0.391	0.996	0.988	1.004
Malignant neoplasm of kidney, except renal pelvis	IVW	15	0.408	1.002	0.996	1.008
Malignant neoplasm of esophagus	IVW	8	0.362	0.997	0.993	1.002
Pancreatic cancer	IVW	3	0.412	1.040	0.946	1.143
Colorectal cancer	IVW	69	0.550	1.005	0.988	1.022

## Data Availability

Data supporting the findings of this study are available from the corresponding author upon reasonable request.
